# Relationships and Mendelian Randomization of Gut Microbe-Derived Metabolites with Metabolic Syndrome Traits in the METSIM Cohort

**DOI:** 10.3390/metabo14030174

**Published:** 2024-03-20

**Authors:** Sahereh Mirzaei, Holli A. DeVon, Rita M. Cantor, Arjen J. Cupido, Calvin Pan, Sung Min Ha, Lilian Fernandes Silva, James R. Hilser, Jaana Hartiala, Hooman Allayee, Federico E. Rey, Markku Laakso, Aldons J. Lusis

**Affiliations:** 1 Department of Medicine, Division of Cardiology, David Geffen School of Medicine, University of California, Los Angeles, CA 90055, USA; 2 School of Nursing, University of California, Los Angeles, CA 90095, USA; 3 Department of Human Genetics, David Geffen School of Medicine, University of California, Los Angeles, CA 90095, USA; 4 Department of Vascular Medicine, Amsterdam University Medical Centers, University of Amsterdam, Amsterdam Cardiovascular Sciences, 1007 AZ Amsterdam, The Netherlands; 5 Department of Integrative Biology and Physiology, University of California, Los Angeles, CA 90095, USA; 6 Department of Clinical Medicine, Internal Medicine, University of Eastern Finland, 70210 Kuopio, Finland; 7 Department of Population & Public Health Sciences, Keck School of Medicine, University of Southern California, Los Angeles, CA 90032, USA; 8 Department of Biochemistry & Molecular Medicine, Keck School of Medicine, University of Southern California, Los Angeles, CA 90033, USA; 9 Department of Bacteriology, University of Wisconsin-Madison, Madison, WI 53706, USA; 10 Department of Human Genetics and Microbiology, Immunology & Molecular Genetics, David Geffen School of Medicine, University of California, Los Angeles, CA 90095, USA

**Keywords:** metabolic syndrome, gut microbes, gut metabolites, insulin resistance, Mendelian randomization, GWAS

## Abstract

The role of gut microbe-derived metabolites in the development of metabolic syndrome (MetS) remains unclear. This study aimed to evaluate the associations of gut microbe-derived metabolites and MetS traits in the cross-sectional Metabolic Syndrome In Men (METSIM) study. The sample included 10,194 randomly related men (age 57.65 ± 7.12 years) from Eastern Finland. Levels of 35 metabolites were tested for associations with 13 MetS traits using lasso and stepwise regression. Significant associations were observed between multiple MetS traits and 32 metabolites, three of which exhibited particularly robust associations. N-acetyltryptophan was positively associated with Homeostatic Model Assessment for Insulin Resistant (HOMA-IR) (β = 0.02, *p* = 0.033), body mass index (BMI) (β = 0.025, *p* = 1.3 × 10^−16^), low-density lipoprotein cholesterol (LDL-C) (β = 0.034, *p* = 5.8 × 10^−10^), triglyceride (0.087, *p* = 1.3 × 10^−16^), systolic (β = 0.012, *p* = 2.5 × 10^−6^) and diastolic blood pressure (β = 0.011, *p* = 3.4 × 10^−6^). In addition, 3-(4-hydroxyphenyl) lactate yielded the strongest positive associations among all metabolites, for example, with HOMA-IR (β = 0.23, *p* = 4.4 × 10^−33^), and BMI (β = 0.097, *p* = 5.1 × 10^−52^). By comparison, 3-aminoisobutyrate was inversely associated with HOMA-IR (β = −0.19, *p* = 3.8 × 10^−51^) and triglycerides (β = −0.12, *p* = 5.9 × 10^−36^). Mendelian randomization analyses did not provide evidence that the observed associations with these three metabolites represented causal relationships. We identified significant associations between several gut microbiota-derived metabolites and MetS traits, consistent with the notion that gut microbes influence metabolic homeostasis, beyond traditional risk factors.

## 1. Introduction

Metabolic syndrome (MetS) is a heterogenous disorder defined by the presence of three or more of the following traits: abdominal obesity, elevated triglycerides, reduced high-density lipoprotein cholesterol (HDL-C), prehypertension or hypertension, and insulin resistance or diabetes mellitus [1]. It is strongly associated with an increased risk for atherosclerosis and other cardiovascular diseases (CVDs) [2,3] contributing to cardiovascular morbidity and mortality in the general population. Having MetS doubles the risk of adverse outcomes and increases by 1.5 fold the risk of mortality from all causes [4]. In addition to CVD and type 2 diabetes (T2D), MetS is associated with the development of non-alcoholic fatty liver disease, cancer, and autoimmune disorders [2,5]. The prevalence of MetS increases with age, from approximately one in five in young adults 20–39, to nearly half of all people over 60 [6,7]. Intestinal flora have been proposed as one of the factors influencing the development of certain MetS traits [8]. The human intestine contains approximately 100 trillion microorganisms and up to 1000 different species of symbiotic bacteria, viruses, archaea, and fungi, collectively termed the gut microbiome [9]. It is influenced by complex interactions involving diet, lifestyle, and host genetics, including crosstalk between intestinal microbes and the host’s immune system [10,11,12].

Although causal relationships between gut microbial profiles and MetS traits have been established in animal studies, the association between the gut microbiota and MetS in humans is still not clearly understood [13]. Gut microbes have a key role in the biosynthesis of numerous metabolites, including amino acid metabolites, which are derived from microbial degradation, and they exhibit extensive effects on the onset of diabetes and metabolic disorders [14]. We found novel associations between amino acid-derived metabolites, including N-acetyltryptophan, 3-(4-hydroxyphenyl) lactate, and 3-aminoisobutyrate, and metabolic syndrome traits; these are metabolites that have been less well studied. 

Approximately 10% of all circulating metabolites in humans are derived from bacteria and participate in various human metabolic or inflammatory pathways [15], contributing to diseases such as atherosclerosis, insulin resistance, and obesity [16]. Although some of the gut microbe-derived metabolites, such as trimethylamine N-oxide (TMAO), have been investigated in depth in the context of CVDs, the relationship between MetS traits and gut microbe-derived metabolites, such as 4-hydroxyphenylacetate, or secondary bile acids, such as glycocholenate sulfate, have rarely been studied. The aim of the present study was to investigate the association between gut microbe-derived metabolites and MetS traits, and to explore the potential causality of the associations in order to identify novel microbial biomarkers for targeted interventions.

## 2. Materials and Methods

### 2.1. The Study Design and Sample Population

This was a secondary analysis of data from a population-based cohort of the Metabolic Syndrome In Men (METSIM) study. The National Cholesterol Education Program Adult Treatment Panel III defined metabolic syndrome criteria for men as follows: waist circumference ≥ 102 cm, blood pressure ≥ 130/85 mmHg, fasting triglyceride levels ≥ 150 mg/dL, fasting HDL-C < 40 mg/dL, and fasting blood sugar ≥ 100 mg/dL [1].

The randomly selected METSIM cohort consists of 10,194 men, aged 45–74, from the population register of Kuopio town, Eastern Finland. Data were collected from 2005 to 2010. Individuals with previously diagnosed diabetes were excluded. Only men were included in the sample because of the influence of sex hormones and the host genetic profile which influence health and disease susceptibility via gut microbial composition. Sex hormones directly modulate the metabolism of bacteria through steroid receptors [17] and their levels are correlated with diversity and gut microbial composition [18]. Host genetics have been linked to differences in microbial composition, suggesting that host genetics can shape the gut microbiome [19]. Finally, the prevalence and incidence of T2D and coronary heart disease are higher in Finnish men than in Finnish women. The cohort underwent detailed phenotyping, particularly for cardiovascular and diabetes-related traits, and had a long follow-up period of 16.6 years. This study extends the impact of the parent study, which aimed to investigate nongenetic and genetic factors associated with MetS and CVD in both cross-sectional and longitudinal analyses. The relatively homogenous nature of the cohort makes it particularly useful for understanding host-microbiome relationships. The METSIM study has been previously described in detail [20].

### 2.2. Setting, Data Collection, and Metabolite Analysis

All patients had a one-day outpatient visit at the University of Kuopio, which included collection of clinical data, health status, and medical treatments. All study participants gave written informed consent. Measures of height, weight, waist, hip, blood pressure (BP), and fat percentages were taken. Fasting blood samples were drawn in the clinic and stored at −80 °C. The following were obtained after 12 h of fasting: glucose, insulin, lipids, lipoproteins, apolipoproteins, adiponectin, bile acids, high sensitivity C-reactive protein, hemoglobin A1c (HbA1c), mass spectrometry metabolomics (Metabolon, Durham, NC, USA), and proton nuclear magnetic resonance (NMR) measurements (lipids and lipoproteins, amino acids, fatty acids of different lengths, and other low-molecular-weight metabolites). Metabolites were measured using Metabolon’s untargeted Discovery HD4 platform using ultra-high-performance liquid chromatography–tandem mass spectroscopy, which has been previously described in detail [21,22]. We selected 35 gut microbe-derived metabolites based on their availability in the METSIM cohort. We excluded metabolites with more than 20% missing values. In addition, based on our literature review, very few studies were available for several metabolites. For example, there was limited literature on the association of 4-hydroxyphenylacetate and methyl indole-3-acetate with metabolic disorders. There are some contradictory findings between studies’ results for some metabolites, suggesting the need for more investigations. For instance, the evidence supporting succinate as a harmful or a beneficial metabolite is inconclusive [23].

### 2.3. Statistical Analysis 

Statistical analyses were conducted using R Studio with R version (4.0.5). We examined the association of 35 gut microbe-derived metabolites with 13 traits including glucose, insulin, Homeostatic Model Assessment for Insulin Resistant (HOMA-IR), hemoglobin A1c (HbA1c), fat mass, body mass index (BMI), waist-to-hip ratio (WHR), low-density lipoprotein cholesterol (LDL-C), high-density lipoprotein cholesterol (HDL-C), total cholesterol, triglycerides, systolic blood pressure (SBP), and diastolic blood pressure (DBP). All metabolites and skewed traits were natural logarithmic transformed. For values under the detectable limit (reported as zeros), we replaced zeros with half of the minimum detectable value of the corresponding metabolite prior to transformation. The relationships between metabolites and traits were assessed using machine learning-based least absolute shrinkage and selection operator (Lasso) regression analysis with a lambda that gave a minimum mean error from a 10-fold cross validation in order to control for overfitting. This provides a conservative estimate of model performance. It has been demonstrated that Lasso is consistent in terms of prediction [24] and variable selection [25], and reduces multicollinearity of metabolic features and retains metabolites with nonzero coefficients [26]. Lasso regression of metabolites allowed us to predict a binary clinical diagnosis when the number of metabolites was very large. It provided a first step in our analysis, as were primarily interested in identifying the individual relative predictive strengths of the salient metabolites, as identified using significant nonzero coefficients in the Lasso regression. Stepwise regression then allowed us to refine our Lasso analysis and identify the most significant predictive metabolites for further evaluation. Metabolites selected using Lasso were included in the stepwise regression models to further evaluate significance of Lasso-selected metabolites, while adjusting for age, BMI, smoking status (yes, no, ex-smoker), exercise (1—a little or none, 2—physical exercise in context of other hobbies or physical exercise, occasionally 3—physical exercise regularly ≤2 times a week at least 30 min at a time, 4—physical exercise regularly ≥3 times a week at least 30 min at a time), daily alcohol consumption (grams per week), and technical covariates including batch effect, regular medication use (yes/no), and the number of medications. 

### 2.4. Mendelian Randomization (MR) Analysis

MR analysis was used to determine whether associations of gut microbe-derived metabolites and metabolic traits represented causal relationships. To maximize the sample size and to use independent datasets, we performed two-sample MR using inverse-variance weighting (IVW), weighted median (WM), and MR-Egger regression methods, as implemented in the two-sample MR package [27,28] in R v4.2.2. The results of a large metabolomics genome-wide association study (GWAS) meta-analysis with the INTERVAL and EPIC-Norfolk cohorts [29] were used to select genetic instrumental variables for gut microbiome metabolites. Variants associated with each metabolite at the genome-wide significance threshold of *p* = 5.0 × 10^−8^ were selected and pruned using clumping (r2 = 0.001) to exclude single nucleotide polymorphisms (SNPs) that were in linkage disequilibrium (LD) with each other. This filtering strategy identified eight variants at four loci that could be used as instrumental variables for 3-aminoisobutyrate, four variants at 3 loci for 3-(4-hydroxyphenyl)lactate, and three variants in three loci for N-acetyltryptophan. Further QC steps during data harmonization were performed to remove palindromic SNPs, which led to the exclusion of one instrumental variable for N-acetyltryptophan. This approach led to the identification of valid instrumental variables that met three key assumptions in MR studies [30], such as being associated with risk factor of interest (relevance assumption), sharing no common cause with the outcome (independence assumption), and not affecting the outcome except through the risk factor (exclusion restriction assumption). Effect estimates for MetS traits as outcomes were taken from published GWAS for BMI, WHR [31], blood lipid levels [32], glycemic traits [33,34], and systolic and diastolic blood pressure [35]. Sensitivity analyses were also performed to detect potential horizontal pleiotropy and directionality of the causal associations using MR Egger intercept and MR Steiger tests, respectively.

## 3. Results

**Figure 1:**
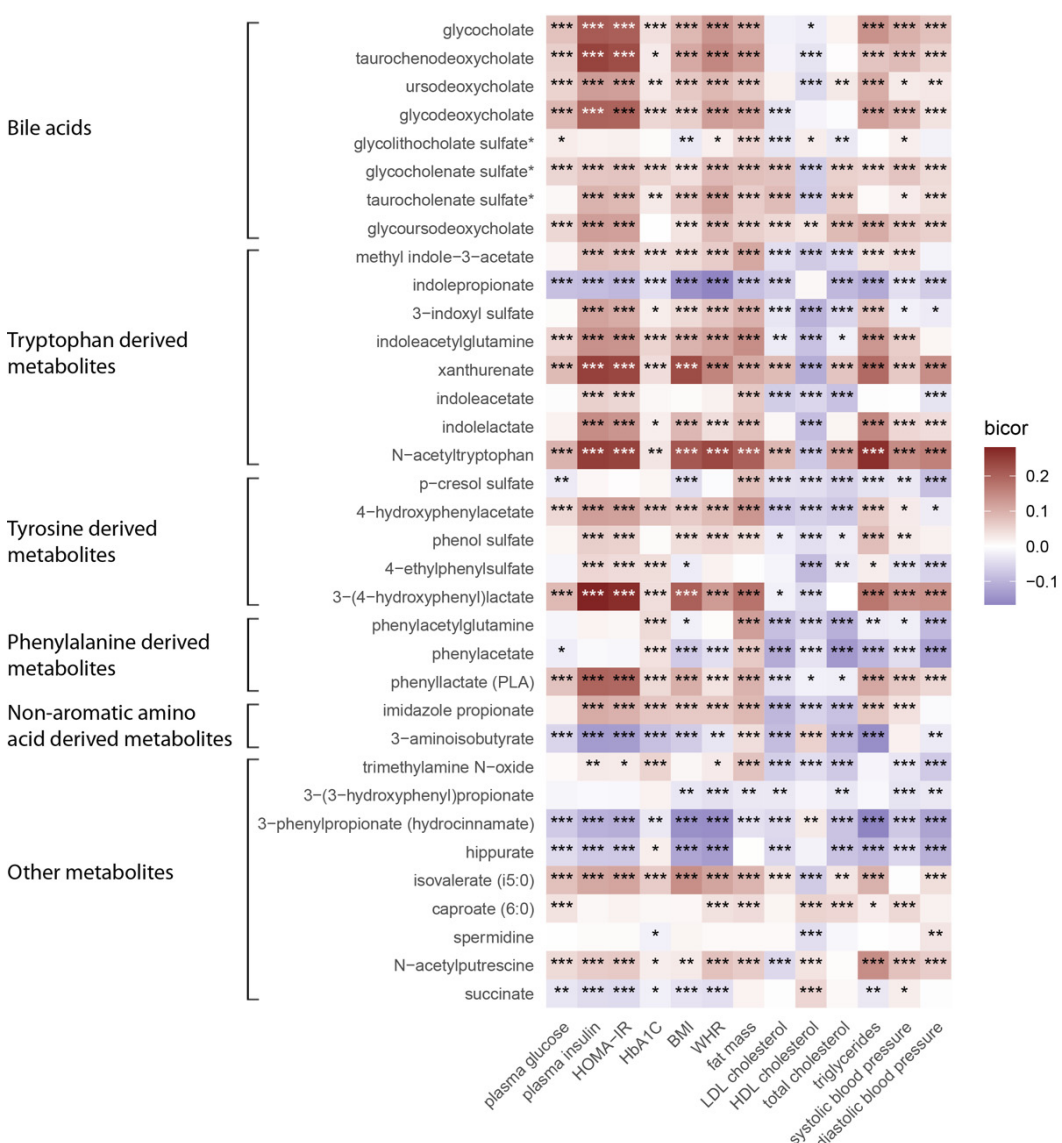
Pearson’s correlation heatmap showing correlations of gut microbe-derived metabolites and metabolic syndrome traits (n = 10,194). Metabolic syndrome traits are shown on x-axis whereas metabolites are displayed on y-axis. Purple color stands for inverse correlations. Red color denotes positive correlations. HOMA-IR, homeostatic model assessment of insulin resistance; HbA1C, hemoglobin A1c; BMI, body mass index; WHR, waist-to-hip ratio; LDL, low-density lipoprotein cholesterol; HDL, high-density lipoprotein cholesterol; * *p*.val < 0.05, ** *p*.val < 0.01, *** *p*.val < 0.001.

**Figure 2:**
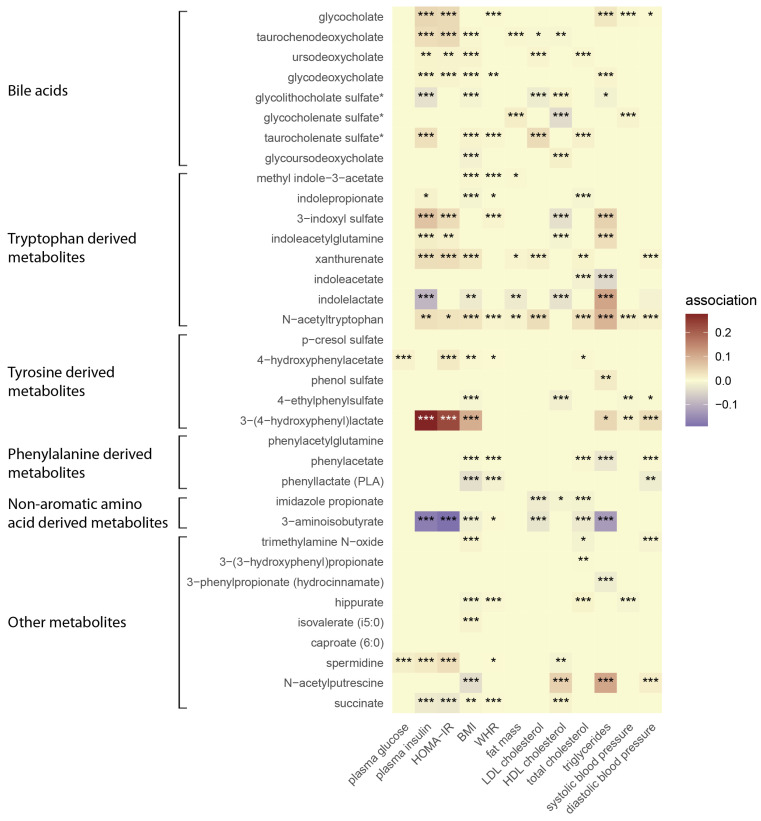
Heatmap showing association between gut microbe-derived metabolites and metabolic symptom traits after adjustments for age, BMI (body mass index), physical activity, medication use, batch effect, alcohol consumption, and smoking. Metabolic syndrome traits are shown on x-axis whereas metabolites are displayed on y-axis. Purple color stands for inverse association. Red color denotes positive associations. HOMA-IR, homeostatic model assessment of insulin resistance; BMI, body mass index; WHR, waist-to-hip ratio; LDL, low-density lipoprotein cholesterol; HDL, high-density lipoprotein cholesterol; data were analyzed using Lasso and stepwise regression. * *p*.val < 0.05, ** *p*.val < 0.01, *** *p*.val < 0.001.

The participants in the present study consisted of 10,194 men with a mean age of 57.65 years. The clinical and laboratory characteristics of the participants are shown in Table 1. We investigated 35 gut microbe-derived metabolites for associations with 13 MetS traits in METSIM and identified several novel associations. Figure 1 shows the correlation heatmap between microbe-derived metabolites and MetS traits. The pairwise correlation heatmap of plasma microbe-derived metabolites is shown in Figure S1. An additional statistical evaluation using Lasso and stepwise regression revealed significant associations between multiple MetS traits and 32 of the 35 metabolites (Figure 2). Of these, N-acetyltryptophan, 3-(4-hydroxyphenyl) lactate, and 3-aminoisobutyrate exhibited particularly robust associations. With the exception of HDL-C, N-acetyltryptophan was positively associated with all MetS traits, including plasma levels of total cholesterol (β = 0.03, *p* = 2.3 × 10^−14^), LDL-C (β = 0.034, *p* = 5.8 × 10^−10^), triglycerides (β = 0.087, *p* = 1.3 × 10^−16^), BMI (β = 0.025, *p* = 1.3 × 10^−16^), WHR (β = 0.005, *p* = 3.0 × 10^−7^), fat mass (β = 0.01, *p* = 0.007), insulin levels (β = 0.032, *p* = 0.005), HOMA-IR (β = 0.02, *p* = 0.033), and systolic (β = 0.012, *p* = 2.5 × 10^−6^) and diastolic blood pressure (DBP) (β = 0.011, *p* = 3.4 × 10^−6^). 

In addition, 3-(4-hydroxyphenyl) lactate yielded the strongest positive associations among all metabolites, including BMI (β = 0.097, *p* = 5.1 × 10^−52^), insulin levels (β = 0.27, *p* = 1.8 × 10^−41^), HOMA-IR (β = 0.23, *p* = 4.4 × 10^−33^), and DBP (β = 0.034, *p* = 2.8 × 10^−12^). The metabolite 3-aminoisobutyrate was inversely associated with plasma insulin (β = −0.17, *p* = 3.6 × 10^−51^), HOMA-IR (β = −0.19, *p* = 3.8 × 10^−51^), serum triglycerides (β = −0.12, *p* = 5.9 × 10^−36^), and total cholesterol (β = −0.022, *p* = 1.3 × 10^−8^).

Among the eight secondary bile acids, only glycolithocholate sulfate was inversely associated with MetS traits including the LDL-C (β = −0.018, *p* = 1.8 × 10^−13^), triglycerides (β = −0.011, *p* = 0.017), and insulin level (β = −0.032, *p* = 3.8 × 10^−8^), BMI (β = −0.008, *p* = 9.9 × 10^−8^). Glycolithocholate sulfate was positively associated with the HDL-C level (β = 0.009, *p* = 3.2 × 10^−4^) (Table S1). 

**Figure 3:**
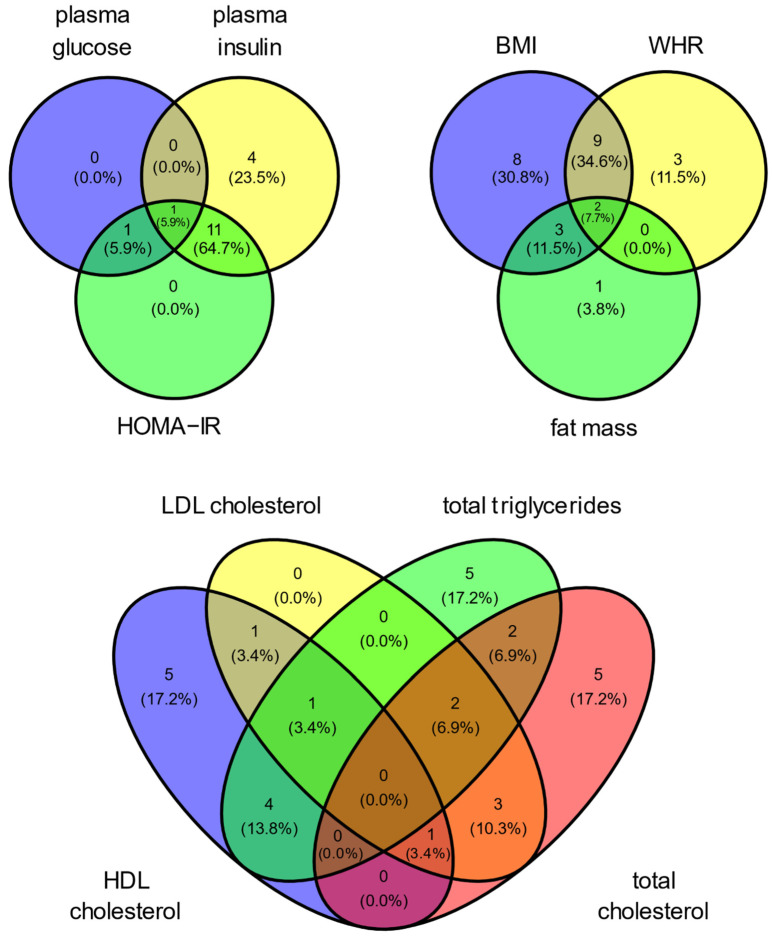
The Venn diagrams are representations of metabolites common to metabolic syndrome traits based on stepwise regression models. Each circle indicates the total number of metabolites associated with the specific trait. The overlapping regions represent the number of metabolites shared between those traits. HOMA-IR, homeostatic model assessment of insulin resistance; BMI, body mass index; WHR, waist-to-hip ratio; LDL, low-density lipoprotein cholesterol; HDL, high-density lipoprotein cholesterol.

Venn diagrams (Figure 3) show the metabolites common to MetS traits. The tryptophan-derived metabolites N-acetyltryptophan and methyl-indole-3-acetate were positively associated with three variables associated with weight including BMI (β = 0.025, *p* = 1.3 × 10^−16^; β = 0.004, *p* = 5.7 × 10^−5^), WHR (β = 0.005, *p* = 3.0 × 10^−7^; β = 0.001, *p* = 4.7 × 10^−4^), and fat mass (β = 0.01, *p* = 0.007; β = 0.003, *p* = 0.011). Spermidine was significantly associated with higher levels of plasma glucose (β = 0.013, *p* = 2.9 × 10^−19^), plasma insulin (β = 0.019, *p* = 1.5 × 10^−4^), and HOMA-IR (β = 0.033, *p* = 4.9 × 10^−9^). 

Phenylalanine-derived metabolites including phenylacetate and phenyllactate were inversely associated with BMI (β = −0.004, *p* = 6.5 × 10^−5^; β = −0.35, *p* = 6.12 × 10^−8^), WHR (β = −0.001, *p* = 3.7 × 10^−4^; β = −0.011, *p* = 8.9 × 10^−14^), and DBP (β = −0.003, *p* = 2.4 × 10^−6^; β = −0.016, *p* = 0.001). 

We next carried out several types of two-sample MR analyses to evaluate whether the observed metabolite–MetS trait associations were causal in nature. We focused these analyses on 3-(4-hydroxyphenyl) lactate, 3-aminoisobutyrate, and N-acetyltryptophan, since the most significant clinical associations were observed with these three gut microbe-derived metabolites. To maximize the power and the number of instrumental variables, we also used publicly available GWAS results for 3-(4-hydroxyphenyl) lactate, 3-aminoisobutyrate, and N-acetyltryptophan derived from a large analysis of the INTERVAL and EPIC-Norfolk cohorts (n = 14,296) [29]. 

At a Bonferroni-corrected threshold for the number of MR tests carried out, IVW and WM MR analyses yielded significant evidence that 3-(4-hydroxyphenyl)lactate is causally associated with triglyceride, total cholesterol, and LDL cholesterol levels, but not with HDL levels or any other selected metabolic outcomes (Table S2). By comparison, MR Egger regression tests did not yield significant results (Table S2). Therefore, we further assessed the robustness of the findings through sensitivity analyses that evaluated the horizontal pleiotropy and directionality with the MR Egger intercept and Steiger’s tests, respectively. These data did not provide evidence for the presence of horizontal pleiotropy (Table S2) and suggested that the direction of the causal association between 3-(4-hydroxyphenyl)lactate and triglyceride, total cholesterol, and LDL cholesterol levels was correct. By comparison, evidence from the MR analyses for N-acetyltryptophan and 3-aminoisobutyrate provided only weak and nominally significant evidence for these metabolites being causally associated with metabolic outcomes (Table S2).

## 4. Discussion

A number of significant associations were identified between microbe-derived metabolites and MetS traits. Aromatic amino acid metabolism by the gut microbiota produces numerous metabolites that may affect the host’s physiology both locally and in other organs [36]. N-acetyltryptophan was significantly associated with nearly all MetS traits including lipid levels, insulin resistance, obesity, and blood pressure. Consistent with our results, Wang et al., in the prospective cohort of China Cardiometabolic Disease and Cancer Cohort (4C) study, found that N-acetyltryptophan was positively associated with incident T2D. In addition, the N-acethltryptophan and T2D association was mediated by triglycerides and the WHR at a proportion of 12% [37]. Bajaj et al. found that N-acetyltryptophan was associated with nosocomial infections in patients with cirrhosis. In addition, N-acetyltrytphan was associated with major negative outcomes including a higher rate of admission, longer hospital length of stays, more frequent transfer to the ICU, organ failure, and death [38]. Huang et al. analyzed longitudinal metabolites from the Cancer Prevention Study, a cohort of 620 men free of CVD who were followed for 28 years. Of the 406 metabolites, a strong association of N-acetyltryptophan (HR = 1.24, *p* = 1.38 × 10^−4^) with all-cause mortality was observed [39].

The gut microbe-derived metabolite 3-(4-hydroxyphenyl)lactate had the strongest positive association with insulin levels (β = 0.27, *p* = 1.8 × 10^−41^) and HOMA-IR (β = 0.23, *p* = 4.4 × 10^−33^. Similarly, one study found that 3-(4-hydroxyphenyl)lactate was significantly associated with diabetes incidence [40]. Another study explored a metabolic profile with weight loss in metabolically healthy obese women after a lifestyle intervention. The authors found that 3-(4-hydroxyphenyl)lactate was positively correlated with several weight variables including weight, BMI, waist, hip, and fat mas in the high weight loss group (>10%) compared to the low weight loss group (<10%) [41]. Our study is the first to report the association between 3-(4-hydroxyphenyl)lactate, triglycerides, and blood pressure. Caussy et al. showed that 3-(4-hydroxyphenyl)lactate is associated with advanced fibrosis in nonalcoholic fatty liver disease (NAFLD) [42]. Hypertriglyceridemia and insulin resistance are common findings in NAFLD patients [43]. Triglyceride levels may be affected by 3-(4-hydroxyphenyl)lactate indirectly through its association with insulin resistance. Chronic exposure to insulin drives very-low-density lipoprotein overproduction which leads to hypertriglyceridemia [44].

Patients with T2D had higher levels of xanthurenate, which was associated with insulin resistance and increased odds of having diabetes [45,46]. Xanthurenate levels have previously been shown to be elevated in obesity [47] and CVD [48]. Similarly, Eussen et al. observed that kynurenine levels, including xanthurenate, were generally higher in participants who had hypertension, were overweight, and who had prediabetes or diabetes [49]. 4-hydroxyphenylacetate and 3-indoxyl sulfate were significantly associated with insulin resistance, obesity, and dyslipidemia. There have been very few studies examining the role of 4-hydroxyphenylacetate in metabolic disorders. Indoxyl sulfate was elevated in a diabetic compared to a nondiabetic control [50]. It has been shown to be associated with nephrotoxicity [51] and there is evidence that it may contribute to the pathogenesis of CVD in chronic kidney disease [52]. In high fat- and high sugar-fed mice, indoxyl sulfate was strongly correlated with all metabolic parameters including plasma glucose, insulin, HOMA-IR, LDL-C, and total cholesterol [53].

Variations in polyamine levels have been associated with multiple diseases including stroke, inflammation, and diabetes [54]. We found significant positive associations between spermidine and insulin resistance and WHR, and an inverse association with HDL-C. A spermidine supplementation resulted in enhanced diabetes incidence in nonobese diabetic mice with an increased proportion of proinflammatory T-cells in pancreatic lymph nodes [55]. In a cross-sectional study with 4230 individuals, serum spermidine was positively associated with increased odds of obesity but reduced odds of an increase in BMI in a follow-up study [56]. N-acetylputrescine was associated with higher levels of triglycerides and HDL-C. Similarly, Hong et al. found that SNP rs35570672-T, which is associated with hyperlipidemia, was also associated with elevated levels of N-acetylputrescine [57]. Increased levels of N-acetylputrescine have been associated with disease severity in patients with liver and kidney disease [58]. Indoleacetylglutamine was significantly associated with insulin resistance, dyslipidemia, major adverse cardiovascular events, and all-cause mortality independent of traditional cardiovascular risk factors in two independent cohorts of subjects undergoing elective diagnostic cardiac evaluation [59].

Indoleacetate, 3-Phenylpropionate, 3-(3-hydroxyphenyl)propionate, and indolepropionate were inversely associated with serum lipids, while phenol sulfate was positively associated with serum lipids. Derivatives of 3-phenylpropionate are known to have agonistic effects on peroxisome proliferator-activated receptors with antidiabetic and lipid-lowering activity [60]. Indole supplementation in mice led to an improvement of metabolic parameters, maintaining the intestinal barrier function and promoting GLP-1 production in one study [61]. Indolepropionate supplementation has been shown to protect against atherosclerotic plaques in apolipoprotein E-deficient mice by promoting macrophage reverse cholesterol transport through the miR-142-5p/ABCA1 signaling pathway [62]. Feng et al. suggest that 3-(3-hydroxyphenyl)propionate has an atheroprotective effect through its inhibition of monocyte adhesion to endothelial cells by modulating the expression of the adhesion molecule E-selectin. This effect is partially mediated by its inhibitory action on the NF-κB activation induced by TNFα [63]. In the present study, phenyllactate was inversely associated with obesity and DBP. It was shown to be an exercise-induced metabolite regulating appetite in a study by Li and colleagues [64]. Phenyllactate has consistently been shown to be higher in undernourished mice than in control mice [65].

The level of 4-Ethylphenyl sulfate was inversely associated with BMI, hypertension, and HDL-C. High plasma levels of 4-ethylphenyl sulfate were associated with a neural axon myelination reduction, impairing oligodendrocyte maturation and reducing the oligodendrocyte–neuron interactions, leading to anxiety-like behavioral characteristics [66]. Elevated levels of 4-ethylphenyl sulfate have been reported in the serum of children with autism spectrum disorders [67].

Phenylacetate was inversely associated with triglycerides, total cholesterol, obesity, and DBP. Phenylacetate could promote the development of NAFLD by inducing triglyceride accumulation in hepatocytes and promoting the expression of lipid metabolism genes. The effect of phenylacetate is part of a multifactorial process informed by the gut microbiome that warrants further studies [68].

Indolelactate was inversely associated with insulin levels and obesity, and positively associated with dyslipidemia. Increased physical activity has been shown to be significantly associated with elevated indolelactate and indolepropionate levels [69]. Qi and colleagues reported that indolelactate was positively and indolepropionate was inversely associated with T2D risk [70]. We observed a significant association between indolelactate and the high triglyceride levels and low HDL-C characteristics of diabetes dyslipidemia. Methyl indole-3-acetate was positively associated with BMI, WHR, and fat mass. Very few studies on methyl indole-3-acetate have been published. A recent study showed that an increase in methyl indole-3-acetate level has a protective effect against ulcerative colitis [71]. Another study in high-fat diet-fed mice reported that a potential prebiotic fiber could increase levels of indole derivatives, including methyl indole-3-acetate, and alleviate cardiac dysfunction [72].

We found that 3-aminoisobutyrate was inversely associated with insulin resistance, obesity, triglycerides, LDL-C, and total cholesterol. Jung et al. indicated that a 3-aminoisobutyrate treatment in mice mitigates insulin resistance, inhibits inflammation, and promotes fatty acid oxidation via the AMP-activated protein kinase (AMPK)-peroxisome proliferator-activated receptor (PPAR)-delta [73]. Moreover, 3-aminoisobutyrate reduces body fat through an increase in fatty acid oxidation and a decrease in hepatic lipogenesis in animals [74,75]. We identified inverse associations between imidazole propionate and LDL-C, HDL-C, and total cholesterol. Van Son et al. examined the association between plasma imidazole propionate levels and HDL-C and LDL-C in non-diabetic overweight/obese individuals. They did not find a correlation between imidazole propionate and LDL-C or HDL-C, perhaps due to a small sample size [76].

Higher blood hippurate levels were associated with a lower BMI, WHR, and SBP, and higher total cholesterol levels. Higher intakes of fruit and whole grains have been shown to be associated with higher levels of hippurate, cross-sectionally and longitudinally. An increase in hippurate was previously associated with reduced odds of having MetS [77]. In the context of high-fat diet-induced obesity, hippurate contributes to metabolic improvements [78].

We found an inverse association between succinate and both insulin resistance and obesity, and a positive association with HDL-C. There is evidence supporting the beneficial effects of intracellular succinate as a modulator of intestinal gluconeogenesis [79] and thermogenesis, which could provide robust protection against diet-induced obesity and improve glucose tolerance [80]. Succinate also acts as a pro-inflammatory stimulus [81] to regulate local stress, the immune response, and tissue damage [82,83,84]. In our study, higher plasma levels of isovalerate were significantly associated with a higher BMI. Studies have shown high levels of isovalerate in obese children [85,86].

At relatively low concentrations, secondary bile acids demonstrate anti-inflammatory actions [87]. However, at high concentrations, they can cause oxidative stress, DNA damage, apoptosis, and mutations [88]. Very few studies on glycocholenate sulfate and taurocholenate sulfate have been published. We found that glycocholenate sulfate was significantly associated with higher fat mass, lower HDL-C, and higher SBP. Glycocholenate sulfate is possibly synthesized from glycine-amidation and the sulfation of 3-beta-hydroxy-5-cholenoic acid. Elevated levels of 3-beta-hydroxy-5-cholenoic acid in patients with primary biliary cirrhosis have been reported [89]. Glycocholenate sulfate has been associated with an increased risk of new onset atrial fibrillation [90]. Increased plasma levels of taurocholenate sulfate were significantly associated with insulin levels, BMI, WHR, LDL-C, and total cholesterol. In one study, a 2–3-fold increase in levels of taurochlenate sulfate was reported in patients with primary dilated cardiomyopathy compared to a control group [91]. Significant associations between ursodeoxycholate and insulin resistance, BMI, and hypercholesterolemia were observed. Ursodeoxycholate has been recognized as a ligand of TGR5, a transmembrane G protein-coupled bile acid receptor that is a key regulator of glucose homeostasis [92]. The administration of ursodeoxycholate decreased glucose levels, increased serum glucagon-like peptide 1 (GLP-1) levels, alleviated hyperinsulinemia, increased the islet areas, and improved islet function in a study by Bai et al. These changes may be related to its roles in enhancing TGR5 gene expression in the intestine, inhibiting the expression of genes in bile acid synthesis, and suppressing liver fibrosis [93]. It has also been reported that ursodeoxycholate lowers glucose levels, LDL-C, and total cholesterol in the context of liver disease. Balan et al. found that ursodeoxycholic acid significantly decreased total cholesterol levels. However, the decrease in cholesterol levels was strongly correlated with serum bilirubin levels (r = 0.70; *p* < 0.001), suggesting the primary lipid-lowering effect occurs via an improvement of the underlying primary biliary cirrhosis [94]. The lipid-lowering action of ursodeoxycholic acid was not the primary outcome in almost all studies in a meta-analysis study [95].

Glycoursodeoxycholate levels were associated with lower BMI and higher HLD-C levels. Interestingly, in a diet-induced obesity mouse model, Sun et al. reported that glycoursodeoxycholate may act as an intestinal farnesoid X receptor (FXR) antagonist, and substantially attenuated body weight gain, and restored glucose intolerance and insulin resistance without disorders in bile acid metabolism and liver injury [96]. In addition, increased serum levels of glycoursodeoxycholate were associated with a decrease in HbA1c and waist circumference in patients with T2D [97]. Taken together, the above evidence suggests a potential beneficial effect of glycoursodeoxycholate in metabolism.

We found that glycolithocholate sulfate was inversely associated with plasma insulin, triglycerides, LDL-C, and BMI, and positively associated with HDL-C. Bagheri et al. evaluated diet quality, plasma metabolites, and the gut microbiome in 150 healthy, lean, and overweight women and men aged 18–50 years. They found that the glycolithocholate sulfate level was higher in participants with higher diet quality. Fatty acid derivatives and amino acids, including branch-chain amino acids, were lower in this group [98]. Weight loss in obese post-menopausal women induced by a commercial very-low-caloric diet (VLCD) improved insulin sensitivity and crown-like structures’ density in subcutaneous adipose tissue, with a decrease in the pro-inflammatory gene profiles. Bagheri et al. found that the metabolite glycolithocholate sulfate increased after VLCD-induced weight loss, which was consistent with altered intestinal bacterial metabolism. Insulin sensitivity, as shown by a reduced HOMA-IR, was improved. A VLCD diet was associated with reduced triglycerides and LDL-C levels [99]. Glycocholate, glycodeoxycholate, and taurochenodeoxycholate were significantly associated with insulin resistance, obesity, and dyslipidemia. Impaired bile acid signaling contributes to insulin resistance through multiple mechanisms, including the FXR and the G protein-coupled bile acid receptor TGR5 [100]. In general, total plasma bile acids levels are positively correlated with obesity, T2D, and NAFLD, as evidenced by higher fasting or postprandial plasma bile acids levels [101]. We found an inverse association between trimethylamine N-oxide (TMAO) and DBP. Higher circulating concentrations of TMAO with aging have been linked to aortic stiffening and increases in systolic but not DBP in 122 healthy adult humans [102].

We also used various forms of MR with instrumental variables derived from analyses with large numbers of subjects to evaluate whether gut microbe-derived metabolites were causally associated with MetS traits. While there was some evidence for 3-(4-hydroxyphenyl)lactate being causally associated with lipids, these results were based on a small number of instrument variables. More notably, the inverse direction of the causal associations predicted using MR analyses between 3-(4-hydroxyphenyl)lactate and lipids were opposite to the positive correlations observed in the clinical analyses. Thus, based on our present MR analyses alone, the causal relationship between 3-(4-hydroxyphenyl)lactate and lipid traits remains inconclusive and will require further evaluation in future studies. The same limitations were also evident in the MR analyses for N-acetyltryptophan and 3-aminoisobutyrate, which would therefore argue for no causal relationship between these metabolites and MetS traits as well. However, since the small number and weak effect sizes of the genetic variants likely led to weak instrument bias, an analysis with larger numbers of appropriate genetic variants will be needed to draw firm conclusions regarding the causal relationship between clinically associated gut microbe-derived metabolites and MetS traits.

The microbial metabolites described above, specifically N-acethyltryptophan, 3-(4-hydroxyphenyl)lactate, and 3-aminoisobutyrate, are implicated in the pathogenesis of metabolic disorders and represent potential biomarkers for the early diagnosis and prognosis of these diseases. An altered gut microbiota results in changes in plasma levels of metabolites prior to the development of clinical symptoms. An early identification of risk and early diagnosis is the most effective way to improve the clinical outcomes and reduce mortality rates.

Future research should focus on the validation of potential metabolite biomarkers identified in this study in a large heterogenous sample, at different time points, for the prevention, diagnosis, and personalized therapeutic targeting of the gut microbiota. Finally, our study identified novel relationships between gut metabolites and clinical traits that can provide direction for future animal studies. Mice could be fed or treated with salient metabolites to explore the development of metabolic disorders.

Lastly, our study had limitations including the cross-sectional nature of the analyses, which limited the ability to make causal inferences regarding the relationship between plasma metabolites with traits of MetS. It is difficult to determine whether MetS followed exposure to metabolites or metabolites were expressed following the development of MetS. For example, does N-acetyltryptophan contribute to hyperlipidemia or does hyperlipidemia cause the expression of N-acetyltryptophan? In addition, only middle-aged and elderly Finnish men were included in this study and whether our findings are generalizable to women, younger persons, and other ethnic and racial groups remains to be determined. Additional research should address the relationship between biological, sex, and other factors that contribute to disparities in MetS and metabolite expression. Diverse samples are needed to guide personalized medicine based on an individual’s genetic makeup.

## 5. Conclusions

In conclusion, we observed strong associations between the gut microbe-derived metabolites and MetS traits. Further mechanistic studies are warranted to validate the observed associations, particularly in more heterogeneous populations.

## Figures and Tables

**Table 1:** Characteristics of participants (n = 10,194).

Clinical Traits	Mean (SD) Total Cohort
Age (Years)	57.65 (7.1)
BMI (Kg/m^2^)	27.30 (4.1)
Waist-to-hip ratio	0.97 (0.07)
Fat mass (%)	24.08 (6.5)
Fasting plasma glucose (mmol/L)	5.98 (1.12)
Fasting plasma insulin (mu/L)	9.94 (12.9)
Hemoglobin A1C (%)	5.80 (0.62)
HOMA-IR	2.88 (5.4)
Serum total cholesterol (mmol/L)	5.30 (1.0)
LDL-C (mmol/L)	3.31 (0.9)
HDL-C (mmol/L)	1.44 (0.4)
Serum total triglyceride (mmol/L)	1.47 (1.0)
Systolic blood pressure (mmHg)	138.29 (16.7)
Diastolic blood pressure (mmHg)	87.31 (9.4)
GFR	0.09 (0.02)
CRP (mg/L)	2.21 (4.5)
Total alcohol/week (gr)	99.9 (135.08)
CHD (family members /relative)	n (%)
No	5919 (58)
Yes, parents’ siblings or cousins but not own parents, siblings, or children	893 (8.8)
Yes, own parents, siblings, or children	3384 (33.2)
Smoke	n (%)
No	4148 (40.7)
Yes	1841 (18.1)
ex-smoker	4205 (41.2)

Data are presented as mean ± SD or n (%). BMI, body mass index; HOMA-IR, homeostatic model assessment of insulin resistance; LDL-C, low-density lipoprotein cholesterol; HDL-C, high-density lipoprotein cholesterol; GFR, glomerular filtration rate; CRP, C-reactive protein; CHD, coronary heart disease.

## Data Availability

The data presented in this study are available on request from M.L. Restrictions apply to the availability of data generated or analyzed during this study to preserve the confidentiality of the participants.

## Supplementary Materials

The following supporting information can be downloaded at: https://www.mdpi.com/article/10.3390/metabo14030174/s1, Figure S1. Shows pairwise correlation heatmap of plasma microbe-derived metabolites. Table S1. Associations between gut microbe-derived metabolites and metabolic traits. Table S2. Results of Mendelian randomization analyses to test causal relationships with three metabolites most strongly associated with clinical traits.

## References

[1] (2001). Expert Panel on Detection, Evaluation, Treatment of High Blood Cholesterol in Adults, Executive Summary of The Third Report of The National Cholesterol Education Program (NCEP) Expert Panel on Detection, Evaluation, and Treatment of High Blood Cholesterol In Adults (Adult Treatment Panel III). JAMA.

[2] Dallmeier D., Larson M.G., Vasan R.S., Keaney J.F., Fontes J.D., Meigs J.B., Fox C.S., Benjamin E.J. (2012). Metabolic syndrome and inflammatory biomarkers: A community-based cross-sectional study at the Framingham Heart Study. Diabetol. Metab. Syndr..

[3] Rochlani Y., Pothineni N.V., Kovelamudi S., Mehta J.L. (2017). Metabolic syndrome: Pathophysiology, management, and modulation by natural compounds. Ther. Adv. Cardiovasc. Dis..

[4] Mottillo S., Filion K.B., Genest J., Joseph L., Pilote L., Poirier P., Rinfret S., Schiffrin E.L., Eisenberg M.J. (2010). The metabolic syndrome and cardiovascular risk a systematic review and meta-analysis. J. Am. Coll. Cardiol..

[5] Rastelli M., Knauf C., Cani P.D. (2018). Gut Microbes and Health: A Focus on the Mechanisms Linking Microbes, Obesity, and Related Disorders. Obesity.

[6] Hirode G., Wong R.J. (2020). Trends in the Prevalence of Metabolic Syndrome in the United States, 2011–2016. JAMA.

[7] Moore J.X., Chaudhary N., Akinyemiju T. (2017). Metabolic Syndrome Prevalence by Race/Ethnicity and Sex in the United States, National Health and Nutrition Examination Survey, 1988–2012. Prev. Chronic Dis..

[8] Sheng S., Yan S., Chen J., Zhang Y., Wang Y., Qin Q., Li W., Li T., Huang M., Ding S. (2022). Gut microbiome is associated with metabolic syndrome accompanied by elevated gamma-glutamyl transpeptidase in men. Front. Cell. Infect. Microbiol..

[9] Rajilic-Stojanovic M., de Vos W.M. (2014). The first 1000 cultured species of the human gastrointestinal microbiota. FEMS Microbiol. Rev..

[10] Festi D., Schiumerini R., Eusebi L.H., Marasco G., Taddia M., Colecchia A. (2014). Gut microbiota and metabolic syndrome. World J. Gastroenterol..

[11] Lozupone C.A., Stombaugh J.I., Gordon J.I., Jansson J.K., Knight R. (2012). Diversity, stability and resilience of the human gut microbiota. Nature.

[12] Lynch S.V., Pedersen O. (2016). The Human Intestinal Microbiome in Health and Disease. N. Engl. J. Med..

[13] Wang P.X., Deng X.R., Zhang C.H., Yuan H.J. (2020). Gut microbiota and metabolic syndrome. Chin. Med. J..

[14] Heiss C.N., Olofsson L.E. (2018). Gut Microbiota-Dependent Modulation of Energy Metabolism. J. Innate Immun..

[15] Moran-Ramos S., Lopez-Contreras B.E., Canizales-Quinteros S. (2017). Gut Microbiota in Obesity and Metabolic Abnormalities: A Matter of Composition or Functionality?. Arch. Med. Res..

[16] Holmes E., Li J.V., Marchesi J.R., Nicholson J.K. (2012). Gut microbiota composition and activity in relation to host metabolic phenotype and disease risk. Cell Metab..

[17] Menon R., Watson S.E., Thomas L.N., Allred C.D., Dabney A., Azcarate-Peril M.A., Sturino J.M. (2013). Diet complexity and estrogen receptor beta status affect the composition of the murine intestinal microbiota. Appl. Environ. Microbiol..

[18] Shin J.H., Park Y.H., Sim M., Kim S.A., Joung H., Shin D.M. (2019). Serum level of sex steroid hormone is associated with diversity and profiles of human gut microbiome. Res. Microbiol..

[19] Bonder M.J., Kurilshikov A., Tigchelaar E.F., Mujagic Z., Imhann F., Vila A.V., Deelen P., Vatanen T., Schirmer M., Smeekens S.P. (2016). The effect of host genetics on the gut microbiome. Nat. Genet..

[20] Laakso M., Kuusisto J., Stancakova A., Kuulasmaa T., Pajukanta P., Lusis A.J., Collins F.S., Mohlke K.L., Boehnke M. (2017). The Metabolic Syndrome in Men study: A resource for studies of metabolic and cardiovascular diseases. J. Lipid Res..

[21] Vangipurapu J., Stancakova A., Smith U., Kuusisto J., Laakso M. (2019). Nine Amino Acids Are Associated With Decreased Insulin Secretion and Elevated Glucose Levels in a 7.4-Year Follow-up Study of 5,181 Finnish Men. Diabetes.

[22] Yin X., Chan L.S., Bose D., Jackson A.U., VandeHaar P., Locke A.E., Fuchsberger C., Stringham H.M., Welch R., Yu K. (2022). Genome-wide association studies of metabolites in Finnish men identify disease-relevant loci. Nat. Commun..

[23] Fernandez-Veledo S., Vendrell J. (2019). Gut microbiota-derived succinate: Friend or foe in human metabolic diseases?. Rev. Endocr. Metab. Disord..

[24] Van de Geer S.A., Bühlmann P. (2009). On the conditions used to prove oracle results for the Lasso. Electron. J. Stat..

[25] Meinshausen N., Bühlmann P. (2006). High-dimensional graphs and variable selection with the Lasso. Ann. Stat..

[26] Klau S., Jurinovic V., Hornung R., Herold T., Boulesteix A.L. (2018). Priority-Lasso: A simple hierarchical approach to the prediction of clinical outcome using multi-omics data. BMC Bioinform..

[27] Hemani G., Zheng J., Elsworth B., Wade K.H., Haberland V., Baird D., Laurin C., Burgess S., Bowden J., Langdon R. (2018). The MR-Base platform supports systematic causal inference across the human phenome. Elife.

[28] Hemani G., Tilling K., Smith G.D. (2017). Orienting the causal relationship between imprecisely measured traits using GWAS summary data. PLoS Genet..

[29] Surendran P., Stewart I.D., Au Yeung V.P.W., Pietzner M., Raffler J., Worheide M.A., Li C., Smith R.F., Wittemans L.B.L., Bomba L. (2022). Rare and common genetic determinants of metabolic individuality and their effects on human health. Nat. Med..

[30] Davies N.M., Holmes M.V., Smith G.D. (2018). Reading Mendelian randomisation studies: A guide, glossary, and checklist for clinicians. BMJ.

[31] Pulit S.L., Stoneman C., Morris A.P., Wood A.R., Glastonbury C.A., Tyrrell J., Yengo L., Ferreira T., Marouli E., Ji Y. (2019). Meta-analysis of genome-wide association studies for body fat distribution in 694 649 individuals of European ancestry. Hum. Mol. Genet..

[32] Graham S.E., Clarke S.L., Wu K.H., Kanoni S., Zajac G.J.M., Ramdas S., Surakka I., Ntalla I., Vedantam S., Winkler T.W. (2021). The power of genetic diversity in genome-wide association studies of lipids. Nature.

[33] Chen J., Spracklen C.N., Marenne G., Varshney A., Corbin L.J., Luan J., Willems S.M., Wu Y., Zhang X., Horikoshi M. (2021). The trans-ancestral genomic architecture of glycemic traits. Nat. Genet..

[34] Lagou V., Magi R., Hottenga J.J., Grallert H., Perry J.R.B., Bouatia-Naji N., Marullo L., Rybin D., Jansen R., Min J.L. (2021). Sex-dimorphic genetic effects and novel loci for fasting glucose and insulin variability. Nat. Commun..

[35] Evangelou E., Warren H.R., Mosen-Ansorena D., Mifsud B., Pazoki R., Gao H., Ntritsos G., Dimou N., Cabrera C.P., Karaman I. (2018). Genetic analysis of over 1 million people identifies 535 new loci associated with blood pressure traits. Nat. Genet..

[36] Liu Y., Hou Y., Wang G., Zheng X., Hao H. (2020). Gut Microbial Metabolites of Aromatic Amino Acids as Signals in Host-Microbe Interplay. Trends Endocrinol. Metab..

[37] Wang S., Li M., Lin H., Wang G., Xu Y., Zhao X., Hu C., Zhang Y., Zheng R., Hu R. (2022). Amino acids, microbiota-related metabolites, and the risk of incident diabetes among normoglycemic Chinese adults: Findings from the 4C study. Cell Rep. Med..

[38] Bajaj J.S., Reddy K.R., Tandon P., Garcia-Tsao G., Kamath P.S., O'Leary J.G., Wong F., Lai J., Vargas H., Thuluvath P.J. (2022). Association of serum metabolites and gut microbiota at hospital admission with nosocomial infection development in patients with cirrhosis. Liver Transpl..

[39] Huang J., Weinstein S.J., Moore S.C., Derkach A., Hua X., Liao L.M., Gu F., Mondul A.M., Sampson J.N., Albanes D. (2018). Serum Metabolomic Profiling of All-Cause Mortality: A Prospective Analysis in the Alpha-Tocopherol, Beta-Carotene Cancer Prevention (ATBC) Study Cohort. Am. J. Epidemiol..

[40] Rebholz C.M., Yu B., Zheng Z., Chang P., Tin A., Kottgen A., Wagenknecht L.E., Coresh J., Boerwinkle E., Selvin E. (2018). Serum metabolomic profile of incident diabetes. Diabetologia.

[41] Palau-Rodriguez M., Garcia-Aloy M., Minarro A., Bernal-Lopez M.R., Brunius C., Gomez-Huelgas R., Landberg R., Tinahones F.J., Andres-Lacueva C. (2020). Effects of a long-term lifestyle intervention on metabolically healthy women with obesity: Metabolite profiles according to weight loss response. Clin. Nutr..

[42] Caussy C., Hsu C., Lo M.T., Liu A., Bettencourt R., Ajmera V.H., Bassirian S., Hooker J., Sy E., Richards L. (2018). Link between gut-microbiome derived metabolite and shared gene-effects with hepatic steatosis and fibrosis in NAFLD. Hepatology.

[43] Machado M., Cortez-Pinto H. (2006). Non-alcoholic steatohepatitis and metabolic syndrome. Curr. Opin. Clin. Nutr. Metab. Care.

[44] Choi S.H., Ginsberg H.N. (2011). Increased very low density lipoprotein (VLDL) secretion, hepatic steatosis, and insulin resistance. Trends Endocrinol. Metab..

[45] Reginaldo C., Jacques P., Scott T., Oxenkrug G., Selhub J., Paul L. (2015). Xanthurenic acid is associated with higher insulin resistance and higher odds of diabetes. Faseb J..

[46] Vangipurapu J., Fernandes Silva L., Kuulasmaa T., Smith U., Laakso M. (2020). Microbiota-Related Metabolites and the Risk of Type 2 Diabetes. Diabetes Care.

[47] Groven N., Reitan S.K., Fors E.A., Guzey I.C. (2021). Kynurenine metabolites and ratios differ between Chronic Fatigue Syndrome, Fibromyalgia, and healthy controls. Psychoneuroendocrinology.

[48] Liu J.J., Movassat J., Portha B. (2019). Emerging role for kynurenines in metabolic pathologies. Curr. Opin. Clin. Nutr. Metab. Care.

[49] Eussen S.J., Ueland P.M., Vollset S.E., Nygard O., Midttun O., Sulo G., Ulvik A., Meyer K., Pedersen E.R., Tell G.S. (2015). Kynurenines as predictors of acute coronary events in the Hordaland Health Study. Int. J. Cardiol..

[50] Suhre K., Meisinger C., Doring A., Altmaier E., Belcredi P., Gieger C., Chang D., Milburn M.V., Gall W.E., Weinberger K.M. (2010). Metabolic footprint of diabetes: A multiplatform metabolomics study in an epidemiological setting. PLoS ONE.

[51] Niwa T., Ise M. (1994). Indoxyl sulfate, a circulating uremic toxin, stimulates the progression of glomerular sclerosis. J. Lab. Clin. Med..

[52] Hung S.C., Kuo K.L., Wu C.C., Tarng D.C. (2017). Indoxyl Sulfate: A Novel Cardiovascular Risk Factor in Chronic Kidney Disease. J. Am. Heart Assoc..

[53] Jiang T., Li Y., Li L., Liang T., Du M., Yang L., Yang J., Yang R., Zhao H., Chen M. (2022). Bifidobacterium longum 070103 Fermented Milk Improve Glucose and Lipid Metabolism Disorders by Regulating Gut Microbiota in Mice. Nutrients.

[54] Park M.H., Igarashi K. (2013). Polyamines and Their Metabolites as Diagnostic Markers of Human Diseases. Biomol. Ther..

[55] Karacay C., Prietl B., Harer C., Ehall B., Haudum C.W., Bounab K., Franz J., Eisenberg T., Madeo F., Kolb D. (2022). The effect of spermidine on autoimmunity and beta cell function in NOD mice. Sci. Rep..

[56] Gao H., Zhang Q., Xu J., Yuan W., Li R., Guo H., Gu C., Feng W., Ma Y., Sun Z. (2022). Elevation of Serum Spermidine in Obese Patients: Results from a Cross-Sectional and Follow-Up Study. Nutrients.

[57] Hong K.U., Walls K.M., Hein D.W. (2023). Non-coding and intergenic genetic variants of human arylamine N-acetyltransferase 2 (NAT2) gene are associated with differential plasma lipid and cholesterol levels and cardiometabolic disorders. Front. Pharmacol..

[58] Mindikoglu A.L., Opekun A.R., Putluri N., Devaraj S., Sheikh-Hamad D., Vierling J.M., Goss J.A., Rana A., Sood G.K., Jalal P.K. (2018). Unique metabolomic signature associated with hepatorenal dysfunction and mortality in cirrhosis. Transl. Res..

[59] Nemet I., Li X.S., Haghikia A., Li L., Wilcox J., Romano K.A., Buffa J.A., Witkowski M., Demuth I., Konig M. (2023). Atlas of gut microbe-derived products from aromatic amino acids and risk of cardiovascular morbidity and mortality. Eur. Heart J..

[60] Menni C., Kastenmuller G., Petersen A.K., Bell J.T., Psatha M., Tsai P.C., Gieger C., Schulz H., Erte I., John S. (2013). Metabolomic markers reveal novel pathways of ageing and early development in human populations. Int. J. Epidemiol..

[61] Natividad J.M., Agus A., Planchais J., Lamas B., Jarry A.C., Martin R., Michel M.L., Chong-Nguyen C., Roussel R., Straube M. (2018). Impaired Aryl Hydrocarbon Receptor Ligand Production by the Gut Microbiota Is a Key Factor in Metabolic Syndrome. Cell Metab..

[62] Xue H., Chen X., Yu C., Deng Y., Zhang Y., Chen S., Chen X., Chen K., Yang Y., Ling W. (2022). Gut Microbially Produced Indole-3-Propionic Acid Inhibits Atherosclerosis by Promoting Reverse Cholesterol Transport and Its Deficiency Is Causally Related to Atherosclerotic Cardiovascular Disease. Circ. Res..

[63] Feng J., Ge C., Li W., Li R. (2022). 3-(3-Hydroxyphenyl)propionic acid, a microbial metabolite of quercetin, inhibits monocyte binding to endothelial cells via modulating E-selectin expression. Fitoterapia.

[64] Li V.L., He Y., Contrepois K., Liu H., Kim J.T., Wiggenhorn A.L., Tanzo J.T., Tung A.S., Lyu X., Zushin P.H. (2022). An exercise-inducible metabolite that suppresses feeding and obesity. Nature.

[65] Preidis G.A., Ajami N.J., Wong M.C., Bessard B.C., Conner M.E., Petrosino J.F. (2016). Microbial-Derived Metabolites Reflect an Altered Intestinal Microbiota during Catch-Up Growth in Undernourished Neonatal Mice. J. Nutr..

[66] Needham B.D., Funabashi M., Adame M.D., Wang Z., Boktor J.C., Haney J., Wu W.L., Rabut C., Ladinsky M.S., Hwang S.J. (2022). A gut-derived metabolite alters brain activity and anxiety behaviour in mice. Nature.

[67] Needham B.D., Adame M.D., Serena G., Rose D.R., Preston G.M., Conrad M.C., Campbell A.S., Donabedian D.H., Fasano A., Ashwood P. (2021). Plasma and Fecal Metabolite Profiles in Autism Spectrum Disorder. Biol. Psychiatry.

[68] Hoyles L., Fernandez-Real J.M., Federici M., Serino M., Abbott J., Charpentier J., Heymes C., Luque J.L., Anthony E., Barton R.H. (2018). Molecular phenomics and metagenomics of hepatic steatosis in non-diabetic obese women. Nat. Med..

[69] Kemppainen S.M., Fernandes Silva L., Lankinen M.A., Schwab U., Laakso M. (2022). Metabolite Signature of Physical Activity and the Risk of Type 2 Diabetes in 7271 Men. Metabolites.

[70] Qi Q., Li J., Yu B., Moon J.Y., Chai J.C., Merino J., Hu J., Ruiz-Canela M., Rebholz C., Wang Z. (2022). Host and gut microbial tryptophan metabolism and type 2 diabetes: An integrative analysis of host genetics, diet, gut microbiome and circulating metabolites in cohort studies. Gut.

[71] Wu P., Yao S., Wang X., Yang L., Wang S., Dai W., Zhang H., He B., Wang X., Wang S. (2023). Oral administration of nanoformulated indoximod ameliorates ulcerative colitis by promoting mitochondrial function and mucosal healing. Int. J. Pharm..

[72] Zhang Z., Liu H., Yu B., Tao H., Li J., Wu Z., Liu G., Yuan C., Guo L., Cui B. (2020). Lycium barbarum polysaccharide attenuates myocardial injury in high-fat diet-fed mice through manipulating the gut microbiome and fecal metabolome. Food Res. Int..

[73] Jung T.W., Hwang H.J., Hong H.C., Yoo H.J., Baik S.H., Choi K.M. (2015). BAIBA attenuates insulin resistance and inflammation induced by palmitate or a high fat diet via an AMPK-PPARdelta-dependent pathway in mice. Diabetologia.

[74] Begriche K., Begriche K., Massart J., Abbey-Toby A., Igoudjil A., Letteron P., Fromenty B. (2008). Beta-aminoisobutyric acid prevents diet-induced obesity in mice with partial leptin deficiency. Obesity.

[75] Shi C.X., Zhao M.X., Shu X.D., Xiong X.Q., Wang J.J., Gao X.Y., Chen Q., Li Y.H., Kang Y.M., Zhu G.Q. (2016). beta-aminoisobutyric acid attenuates hepatic endoplasmic reticulum stress and glucose/lipid metabolic disturbance in mice with type 2 diabetes. Sci. Rep..

[76] van Son J., Serlie M.J., Stahlman M., Backhed F., Nieuwdorp M., Aron-Wisnewsky J. (2021). Plasma Imidazole Propionate Is Positively Correlated with Blood Pressure in Overweight and Obese Humans. Nutrients.

[77] Pallister T., Jackson M.A., Martin T.C., Zierer J., Jennings A., Mohney R.P., MacGregor A., Steves C.J., Cassidy A., Spector T.D. (2017). Hippurate as a metabolomic marker of gut microbiome diversity: Modulation by diet and relationship to metabolic syndrome. Sci. Rep..

[78] Brial F., Chilloux J., Nielsen T., Vieira-Silva S., Falony G., Andrikopoulos P., Olanipekun M., Hoyles L., Djouadi F., Neves A.L. (2021). Human and preclinical studies of the host-gut microbiome co-metabolite hippurate as a marker and mediator of metabolic health. Gut.

[79] Wang K., Liao M., Zhou N., Bao L., Ma K., Zheng Z., Wang Y., Liu C., Wang W., Wang J. (2019). Parabacteroides distasonis Alleviates Obesity and Metabolic Dysfunctions via Production of Succinate and Secondary Bile Acids. Cell Rep..

[80] Mills E.L., Pierce K.A., Jedrychowski M.P., Garrity R., Winther S., Vidoni S., Yoneshiro T., Spinelli J.B., Lu G.Z., Kazak L. (2018). Accumulation of succinate controls activation of adipose tissue thermogenesis. Nature.

[81] Littlewood-Evans A., Sarret S., Apfel V., Loesle P., Dawson J., Zhang J., Muller A., Tigani B., Kneuer R., Patel S. (2016). GPR91 senses extracellular succinate released from inflammatory macrophages and exacerbates rheumatoid arthritis. J. Exp. Med..

[82] Chouchani E.T., Pell V.R., Gaude E., Aksentijevic D., Sundier S.Y., Robb E.L., Logan A., Nadtochiy S.M., Ord E.N.J., Smith A.C. (2014). Ischaemic accumulation of succinate controls reperfusion injury through mitochondrial ROS. Nature.

[83] Murphy M.P., O’Neill L.A.J. (2018). Krebs Cycle Reimagined: The Emerging Roles of Succinate and Itaconate as Signal Transducers. Cell.

[84] Correa P.R., Kruglov E.A., Thompson M., Leite M.F., Dranoff J.A., Nathanson M.H. (2007). Succinate is a paracrine signal for liver damage. J. Hepatol..

[85] Riva A., Borgo F., Lassandro C., Verduci E., Morace G., Borghi E., Berry D. (2017). Pediatric obesity is associated with an altered gut microbiota and discordant shifts in Firmicutes populations. Environ. Microbiol..

[86] Gyarmati P., Song Y., Dotimas J., Yoshiba G., Christison A. (2021). Cross-sectional comparisons of gut microbiome and short-chain fatty acid levels among children with varied weight classifications. Pediatr. Obes..

[87] Ward J.B.J., Lajczak N.K., Kelly O.B., O'Dwyer A.M., Giddam A.K., Ni Gabhann J., Franco P., Tambuwala M.M., Jefferies C.A., Keely S. (2017). Ursodeoxycholic acid and lithocholic acid exert anti-inflammatory actions in the colon. Am. J. Physiol. Gastrointest. Liver Physiol..

[88] Ajouz H., Mukherji D., Shamseddine A. (2014). Secondary bile acids: An underrecognized cause of colon cancer. World J. Surg. Oncol..

[89] Minder E.I., Karlaganis G., Paumgartner G. (1979). Radioimmunological determination of serum 3 beta-hydroxy-5-cholenoic acid in normal subjects and patients with liver disease. J. Lipid Res..

[90] Alonso A., Yu B., Sun Y.V., Chen L.Y., Loehr L.R., O'Neal W.T., Soliman E.Z., Boerwinkle E. (2019). Serum Metabolomics and Incidence of Atrial Fibrillation (from the Atherosclerosis Risk in Communities Study). Am. J. Cardiol..

[91] Alexander D., Lombardi R., Rodriguez G., Mitchell M.M., Marian A.J. (2011). Metabolomic distinction and insights into the pathogenesis of human primary dilated cardiomyopathy. Eur. J. Clin. Investig..

[92] Reich M., Deutschmann K., Sommerfeld A., Klindt C., Kluge S., Kubitz R., Ullmer C., Knoefel W.T., Herebian D., Mayatepek E. (2016). TGR5 is essential for bile acid-dependent cholangiocyte proliferation in vivo and in vitro. Gut.

[93] Bai X.P., Du W.J., Xing H.B., Yang G.H., Bai R. (2023). Influence of ursodeoxycholic acid on blood glucose, insulin and GLP-1 in rats with liver fibrosis induced by bile duct ligation. Diabetol. Metab. Syndr..

[94] Balan V., Dickson E.R., Jorgensen R.A., Lindor K.D. (1994). Effect of ursodeoxycholic acid on serum lipids of patients with primary biliary cirrhosis. Mayo Clin. Proc..

[95] Simental-Mendia L.E., Simental-Mendia M., Sanchez-Garcia A., Banach M., Serban M.C., Cicero A.F.G., Sahebkar A. (2019). Impact of ursodeoxycholic acid on circulating lipid concentrations: A systematic review and meta-analysis of randomized placebo-controlled trials. Lipids Health Dis..

[96] Sun L., Xie C., Wang G., Wu Y., Wu Q., Wang X., Liu J., Deng Y., Xia J., Chen B. (2018). Gut microbiota and intestinal FXR mediate the clinical benefits of metformin. Nat. Med..

[97] Witkin S.S., Moron A.F., Ridenhour B.J., Minis E., Hatanaka A., Sarmento S.G.P., Franca M.S., Carvalho F.H.C., Hamamoto T.K., Mattar R. (2019). Vaginal Biomarkers That Predict Cervical Length and Dominant Bacteria in the Vaginal Microbiomes of Pregnant Women. mBio.

[98] Bagheri M., Shah R.D., Mosley J.D., Ferguson J.F. (2021). A metabolome and microbiome wide association study of healthy eating index points to the mechanisms linking dietary pattern and metabolic status. Eur. J. Nutr..

[99] Aleman J.O., Iyengar N.M., Walker J.M., Milne G.L., Da Rosa J.C., Liang Y., Giri D.D., Zhou X.K., Pollak M.N., Hudis C.A. (2017). Effects of Rapid Weight Loss on Systemic and Adipose Tissue Inflammation and Metabolism in Obese Postmenopausal Women. J. Endocr. Soc..

[100] Shapiro H., Kolodziejczyk A.A., Halstuch D., Elinav E. (2018). Bile acids in glucose metabolism in health and disease. J. Exp. Med..

[101] Chavez-Talavera O., Haas J., Grzych G., Tailleux A., Staels B. (2019). Bile acid alterations in nonalcoholic fatty liver disease, obesity, insulin resistance and type 2 diabetes: What do the human studies tell?. Curr. Opin. Lipidol..

[102] Brunt V.E., Casso A.G., Gioscia-Ryan R.A., Sapinsley Z.J., Ziemba B.P., Clayton Z.S., Bazzoni A.E., VanDongen N.S., Richey J.J., Hutton D.A. (2021). Gut Microbiome-Derived Metabolite Trimethylamine N-Oxide Induces Aortic Stiffening and Increases Systolic Blood Pressure With Aging in Mice and Humans. Hypertension.

