# Supplementary material for: Relationships and Mendelian Randomization of Gut Microbe-Derived Metabolites with Metabolic Syndrome Traits in the METSIM Cohort
Source: Metabolites. 2024 Mar 20;14(3):174. doi: 10.3390/metabo14030174 (PMC10972019; doi:10.3390/metabo14030174)
Supplement: Supplementary file 1 [file metabolites-14-00174-s001.zip › metabolites-2912705-supplementary.pdf]

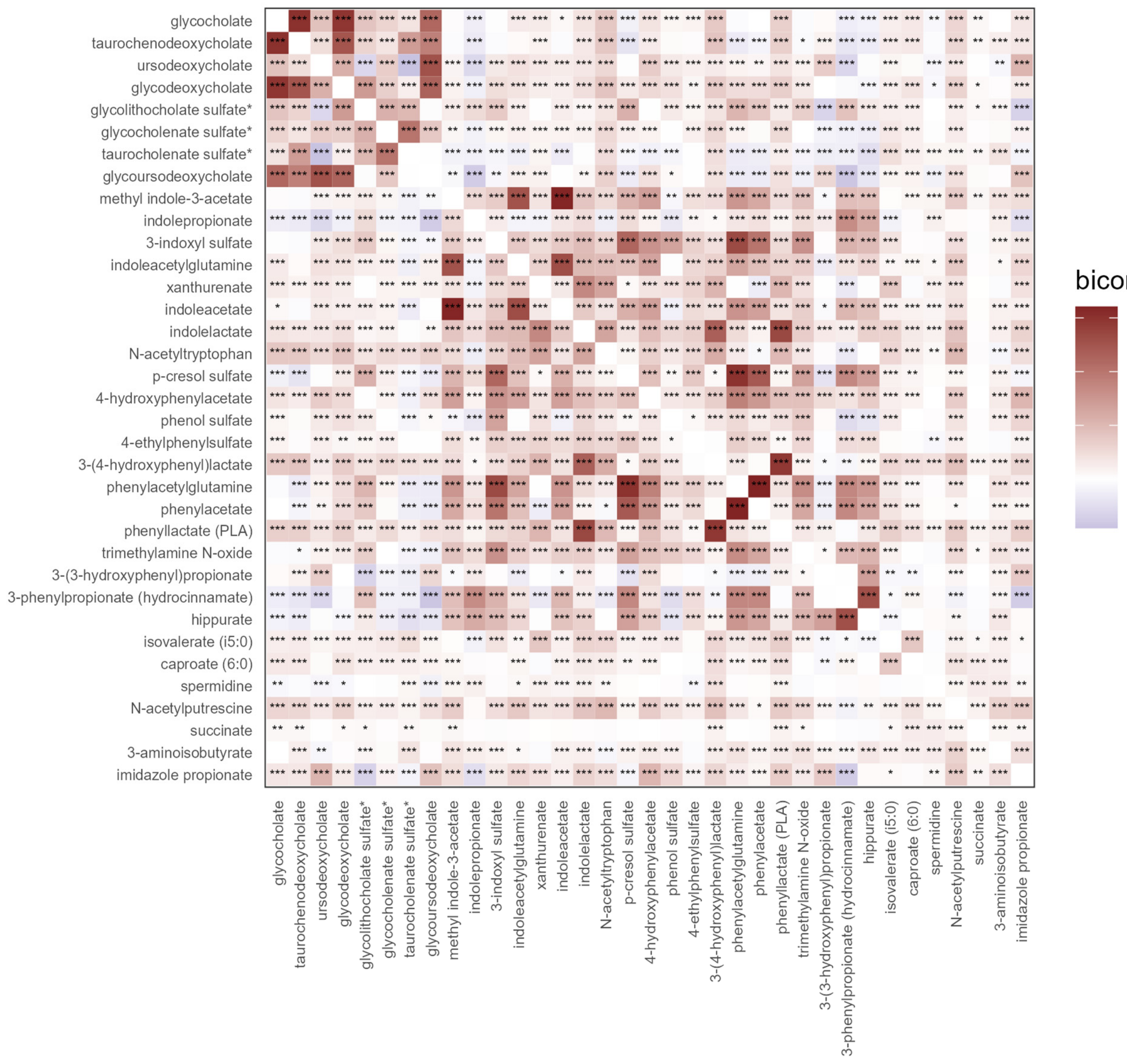

Supplementary Figure S1. Shows pairwise correlation heatmap of plasma microbe-derived metabolites.

Supplementary Table S1. Associations between gut microbe-derived metabolites and metabolic traits.

Gut Derived Metabolites	Insulin			HOMA-IR		
	Beta	SD	P	Beta	SD	P
3-(4-hydroxyphenyl)lactate	0.276	0.020	1.84E-41	0.231	0.019	4.41E-33
N-acetyltryptophan	0.032	0.011	0.005	0.02	0.013	0.033
xanthurenate	0.034	0.007	8.7E-07	0.035	0.007	4.7E-06
3-indoxyl sulfate	0.069	0.008	1.2E-14	0.036	0.009	2.2E-04
spermidine	0.019	0.005	1.5E-04	0.033	0.005	4.9E-09
indoleacetylglutamine	0.015	0.003	8.9E-06	0.010	0.003	0.006
taurochenodeoxycholate	0.035	0.004	4.3E-14	0.040	0.004	1.2E-16
ursodeoxycholate	0.007	0.002	0.003	0.008	0.002	0.004
glycodeoxycholate	0.007	0.001	1.2E-05	0.007	0.001	1.1E-04
glycocholate	0.044	0.006	1.5E-11	0.040	0.007	2.0E-08
3-aminoisobutyrate	-0.170	0.011	3.6E-51	-0.190	0.012	3.8E-51
succinate	-0.020	0.005	4.5E-04	-0.023	0.006	4.2E-04
indolelactate	-0.091	0.022	6.7E-05			
glycolithocholate sulfate*	-0.032	0.005	3.8E-08			
taurocholenate sulfate*	0.032	0.009	2.8E-04			
indolepropionate	0.010	0.004	0.034			
4-hydroxyphenylacetate				0.020	0.005	2.0E-04

Gut Derived Metabolites	HDL-C			LDL-C		
	Beta	SD	P	Beta	SD	P
3-indoxyl sulfate	-0.034	0.004	1.2E-14			
indoleacetylglutamine	-0.008	0.001	2.7E-06			
4-ethylphenylsulfate	-0.014	0.003	2.9E-05			
indolelactate	-0.030	0.009	6.5E-04			
spermidine	-0.007	0.002	0.001			
succinate	0.010	0.002	4.4E-04			
glycocholenate sulfate*	-0.040	0.005	2.5E-12			
N-acetylputrescine	0.051	0.008	2.7E-09			
glycoursodeoxycholate	0.012	0.002	2.1E-08			
glycolithocholate sulfate*	0.009	0.002	3.2E-04	-0.018	0.002	1.8E-13
taurochenodeoxycholate	-0.005	0.001	0.001	-0.004	0.001	0.015
imidazole propionate	-0.007	0.003	0.020	-0.019	0.003	5.2E-09
N-acetyltryptophan				0.034	0.005	5.8E-10
taurocholenate sulfate*				0.038	0.004	4.2E-19
3-aminoisobutyrate				-0.027	0.005	1.09E-06
ursodeoxycholate				0.006	0.001	5.1E-07
xanthurenate				0.012	0.003	1.3E-04

Gut Derived Metabolites	Total cholesterol			Total Triglycerides		
	Beta	SD	P	Beta	SD	P
N-acetyltryptophan	0.030	0.003	2.3E-14	0.087	0.010	1.3E-16
3-aminoisobutyrate	-0.022	0.003	1.3E-08	-0.128	0.010	5.9E-36
phenylacetate	-0.005	0.001	3.5E-04	-0.024	0.004	2.4E-09
indoleacetate	-0.011	0.003	6.2E-04	-0.044	0.009	5.8E-06
ursodeoxycholate	0.005	0.0009	4.9E-08			
taurocholenate sulfate*	0.011	0.002	4.1E-05			
imidazole propionate	-0.009	0.002	9.9E-05			
indolepropionate	-0.006	0.001	4.5E-04			
xanthurenate	0.007	0.002	0.001			
trimethylamine N-oxide	-0.008	0.003	0.012			
hippurate	0.008	0.002	4.7E-04			
3-(3-hydroxyphenyl)propionate	-0.003	0.0009	0.001			
4-hydroxyphenylacetate	0.003	0.001	0.027			
indolelactate				0.108	0.020	7.4E-08
3-phenylpropionate				-0.017	0.003	1.4E-07
indoleacetylglutamine				0.033	0.003	1.3E-20
N-acetylputrescine				0.107	0.016	2.9E-11

glycocholate				0.023	0.004	8.3E-08
phenol sulfate				0.018	0.006	0.007
3-indoxyl sulfate				0.050	0.009	2.5E-07
glycodeoxycholate				0.006	0.001	1.9E-05
3-(4-hydroxyphenyl)lactate				0.045	0.018	0.012
glycolithocholate sulfate*				-0.011	0.005	0.017

**Gut Derived Metabolites**	**Systolic blood pressure**			**Diastolic blood pressure**		
	**Beta**	**SD**	**P**	**Beta**	**SD**	**P**
N-acetyltryptophan	0.012	0.002	2.5E-06	0.011	0.002	3.4E-06
3-(4-hydroxyphenyl)lactate	0.011	0.003	0.002	0.034	0.005	2.8E-12
glycocholate	0.004	0.001	2.3E-05	0.002	0.0009	0.021
4-ethylphenylsulfate	-0.004	0.001	0.005	-0.003	0.001	0.033
hippurate	-0.007	0.001	5.2E-09			
glycocholenate sulfate*	0.008	0.002	7.5E-04			
N-acetylputrescine				0.014	0.003	6.6E-05
xanthurenate				0.006	0.001	1.3E-05
phenylacetate				-0.003	0.0007	2.4E-06
phenyllactate				-0.016	0.005	0.001
trimethylamine N-oxide				-0.008	0.002	3.7E-05

**Gut Derived Metabolites**	**BMI**			**WHR**			**Fat Mass**		
	**Beta**	**SD**	**P**	**Beta**	**SD**	**P**	**Beta**	**SD**	**P**
N-acetyltryptophan	0.025	0.003	1.3E-16	0.005	0.001	3.0E-07	0.010	0.003	0.007
methyl indole-3-acetate	0.004	0.001	5.7E-05	0.001	0.0004	4.7E-04	0.003	0.001	0.011
taurocholenate sulfate*	0.008	0.002	1.9E-04	0.004	0.0007	2.4E-09			
glycodeoxycholate	0.003	0.0004	3.9E-11	0.004	0.0001	0.003			
4-hydroxyphenylacetate	0.003	0.001	0.006	0.001	0.0004	0.025			
3-aminoisobutyrate	-0.014	0.002	5.2E-07	0.002	0.001	0.014			
succinate	-0.004	0.001	0.002	-0.001	0.0005	6.0E-04			
phenylacetate	-0.004	0.001	6.5E-05	-0.001	0.0004	3.7E-04			
phenyllactate	-0.035	0.006	6.1E-08	-0.011	0.001	8.9E-14			
hippurate	-0.007	0.001	3.6E-07	-0.003	0.0005	1.6E-10			
xanthurenate	0.023	0.001	3.8E-36				0.006	0.002	0.012
indolelactate	-0.021	0.006	0.001				-0.020	0.006	0.001
taurochenodeoxycholate	0.004	0.001	9.5E-06				0.005	0.001	5.0E-06
ursodeoxycholate	0.007	0.0008	1.4E-18						
glycoursodeoxycholate	-0.011	0.001	7.0E-17						
N-acetylputrescine	-0.036	0.004	5.0E-15						
isovalerate (i5:0)	0.008	0.001	3.7E-06						
glycolithocholate sulfate*	-0.008	0.001	9.9E-08						
3-(4-hydroxyphenyl)lactate	0.097	0.006	5.1E-52						
indolepropionate	-0.007	0.001	3.4E-08						
4-ethylphenylsulfate	-0.005	0.001	9.8E-04						
trimethylamine N-oxide	0.008	0.002	9.9E-04						
glycocholate				0.002	0.0004	1.3E-09			
spermidine				0.001	0.0004	0.020			
3-indoxyl sulfate				0.005	0.0009	6.7E-09			
Indolepropionate				-0.001	0.0004	0.032			
glycocholenate sulfate*							0.016	0.003	1.2E-05

Supplementary Table S2. Results of Mendelian randomization analyses to test causal relationships with three metabolites most strongly associated with clinical traits.

**Metabolite** (Exposure)	**Outcome**	**Method**	**No. of Instrumental Variables**	**Beta**	**SE**	**p-value**
3-(4-hydroxyphenyl)lactate	TG	WM	4	-0.046	0.010	**<0.0001**
3-(4-hydroxyphenyl)lactate	TG	IVW	4	-0.043	0.019	**0.022**
3-(4-hydroxyphenyl)lactate	TG	MR Egger	4	0.010	0.095	0.927
3-(4-hydroxyphenyl)lactate	TG	Pleiotropy - Egger Intercept		-0.008	0.014	0.622
3-(4-hydroxyphenyl)lactate	TC	WM	4	-0.046	0.010	**<0.0001**
3-(4-hydroxyphenyl)lactate	TC	IVW	4	-0.043	0.019	**0.022**
3-(4-hydroxyphenyl)lactate	TC	MR Egger	4	0.010	0.095	0.927
3-(4-hydroxyphenyl)lactate	TC	Pleiotropy - Egger Intercept		-0.008	0.014	0.622
3-(4-hydroxyphenyl)lactate	LDL	WM	4	-0.037	0.010	**0.00016**
3-(4-hydroxyphenyl)lactate	LDL	IVW	4	-0.038	0.023	0.095
3-(4-hydroxyphenyl)lactate	LDL	MR Egger	4	-0.087	0.118	0.539
3-(4-hydroxyphenyl)lactate	LDL	Pleiotropy - Egger Intercept		0.007	0.017	0.715
3-(4-hydroxyphenyl)lactate	HDL	WM	4	0.009	0.009	0.326
3-(4-hydroxyphenyl)lactate	HDL	IVW	4	0.011	0.013	0.414
3-(4-hydroxyphenyl)lactate	HDL	MR Egger	4	-0.051	0.056	0.458
3-(4-hydroxyphenyl)lactate	HDL	Pleiotropy - Egger Intercept		0.009	0.008	0.373
3-(4-hydroxyphenyl)lactate	BMI	WM	3	-0.028	0.024	0.241
3-(4-hydroxyphenyl)lactate	BMI	IVW	3	-0.030	0.036	0.412
3-(4-hydroxyphenyl)lactate	BMI	MR Egger	3	0.140	0.053	0.231
3-(4-hydroxyphenyl)lactate	BMI	Pleiotropy - Egger Intercept		-0.024	0.007	0.185
3-(4-hydroxyphenyl)lactate	WHR	WM	3	-0.036	0.020	0.068
3-(4-hydroxyphenyl)lactate	WHR	IVW	3	-0.025	0.017	0.139
3-(4-hydroxyphenyl)lactate	WHR	MR Egger	3	0.047	0.054	0.544
3-(4-hydroxyphenyl)lactate	WHR	Pleiotropy - Egger Intercept		-0.010	0.007	0.395
3-(4-hydroxyphenyl)lactate	Glucose	IVW	2	0.038	0.073	0.603
3-(4-hydroxyphenyl)lactate	Insulin	IVW	2	0.019	0.058	0.740
3-(4-hydroxyphenyl)lactate	HbA1c	WM	4	0.000	0.009	0.998
3-(4-hydroxyphenyl)lactate	HbA1c	IVW	4	-0.001	0.009	0.924
3-(4-hydroxyphenyl)lactate	HbA1c	MR Egger	4	-0.009	0.051	0.883
3-(4-hydroxyphenyl)lactate	HbA1c	Pleiotropy - Egger Intercept		0.001	0.008	0.892
3-(4-hydroxyphenyl)lactate	SBP	WM	3	0.501	0.290	0.085
3-(4-hydroxyphenyl)lactate	SBP	IVW	3	0.485	0.242	**0.044**
3-(4-hydroxyphenyl)lactate	SBP	MR Egger	3	0.148	0.852	0.891

3-(4-hydroxyphenyl)lactate	SBP	Pleiotropy - Egger Intercept		0.047	0.113	0.751
3-(4-hydroxyphenyl)lactate	DBP	WM	3	-0.062	0.176	0.724
3-(4-hydroxyphenyl)lactate	DBP	IVW	3	-0.033	0.175	0.848
3-(4-hydroxyphenyl)lactate	DBP	MR Egger	3	0.798	0.489	0.350
3-(4-hydroxyphenyl)lactate	DBP	Pleiotropy - Egger Intercept		-0.115	0.065	0.327
3-aminoisobutyrate	TG	WM	8	-0.001	0.003	0.710
3-aminoisobutyrate	TG	IVW	8	-0.036	0.074	0.625
3-aminoisobutyrate	TG	MR Egger	8	0.131	0.111	0.282
3-aminoisobutyrate	TG	Pleiotropy - Egger Intercept		-0.054	0.029	0.114
3-aminoisobutyrate	TC	WM	8	-0.001	0.003	0.711
3-aminoisobutyrate	TC	IVW	8	-0.036	0.074	0.625
3-aminoisobutyrate	TC	MR Egger	8	0.131	0.111	0.282
3-aminoisobutyrate	TC	Pleiotropy - Egger Intercept		-0.054	0.029	0.114
3-aminoisobutyrate	LDL	WM	8	0.002	0.003	0.438
3-aminoisobutyrate	LDL	IVW	8	-0.007	0.021	0.747
aminoisobutyrate	LDL	MR Egger	8	0.035	0.033	0.324
aminoisobutyrate	LDL	Pleiotropy - Egger Intercept	8	-0.014	0.009	0.168
aminoisobutyrate	HDL	WM	8	0.002	0.003	0.564
aminoisobutyrate	HDL	IVW	8	0.005	0.005	0.355
aminoisobutyrate	HDL	MR Egger	8	-0.009	0.006	0.210
aminoisobutyrate	HDL	Pleiotropy - Egger Intercept		0.004	0.002	**0.041**
aminoisobutyrate	BMI	WM	7	0.002	0.004	0.523
aminoisobutyrate	BMI	IVW	7	0.005	0.007	0.430
aminoisobutyrate	BMI	MR Egger	7	-0.010	0.009	0.306
aminoisobutyrate	BMI	Pleiotropy - Egger Intercept		0.005	0.002	0.094
aminoisobutyrate	WHR	WM	7	-0.005	0.004	0.187
aminoisobutyrate	WHR	IVW	7	-0.006	0.006	0.316
aminoisobutyrate	WHR	MR Egger	7	0.000	0.010	0.979
aminoisobutyrate	WHR	Pleiotropy - Egger Intercept		-0.002	0.003	0.561
aminoisobutyrate	Glucose	WM	5	0.001	0.006	0.911
aminoisobutyrate	Glucose	IVW	5	-0.007	0.029	0.815
aminoisobutyrate	Glucose	MR Egger	5	0.044	0.057	0.502
aminoisobutyrate	Glucose	Pleiotropy - Egger Intercept		-0.018	0.017	0.383
aminoisobutyrate	Insulin	WM	5	0.002	0.007	0.739
aminoisobutyrate	Insulin	IVW	5	0.001	0.018	0.949
aminoisobutyrate	Insulin	MR Egger	5	0.015	0.041	0.738
aminoisobutyrate	Insulin	Egger Intercept		-0.005	0.012	0.722
aminoisobutyrate	HbA1c	WM	8	-0.006	0.003	0.047

aminoisobutyrate	HbA1c	IVW	8	-0.003	0.006	0.593
aminoisobutyrate	HbA1c	MR Egger	8	-0.004	0.011	0.731
aminoisobutyrate	HbA1c	Pleiotropy - Egger Intercept		0.000	0.003	0.930
aminoisobutyrate	SBP	WM	7	-0.073	0.078	0.348
aminoisobutyrate	SBP	IVW	7	-0.163	0.153	0.287
aminoisobutyrate	SBP	MR Egger	7	0.047	0.264	0.864
aminoisobutyrate	SBP	Pleiotropy - Egger Intercept		-0.068	0.069	0.372
aminoisobutyrate	DBP	WM	7	-0.063	0.043	0.140
aminoisobutyrate	DBP	IVW	7	-0.083	0.058	0.152
aminoisobutyrate	DBP	MR Egger	7	0.023	0.092	0.809
aminoisobutyrate	DBP	Pleiotropy - Egger Intercept		-0.034	0.024	0.214
N-acetyltryptophan	TG	IVW	2	0.008	0.005	0.096
N-acetyltryptophan	TC	IVW	2	0.008	0.005	0.096
N-acetyltryptophan	LDL	IVW	2	-0.006	0.005	0.263
N-acetyltryptophan	HDL	IVW	2	-0.011	0.005	**0.027**
N-acetyltryptophan	BMI	IVW	2	0.017	0.007	**0.010**
N-acetyltryptophan	WHR	IVW	2	0.008	0.007	0.251
N-acetyltryptophan	Glucose	IVW	2	0.005	0.012	0.693
N-acetyltryptophan	Insulin	IVW	2	0.028	0.020	0.157
N-acetyltryptophan	HbA1c	IVW	2	0.010	0.005	**0.042**
N-acetyltryptophan	SBP	IVW	2	0.024	0.251	0.923
N-acetyltryptophan	DBP	IVW	2	0.102	0.063	0.110

SE: standard error; TG; triglycerides; TC: total cholesterol; LDL: low density lipoprotein; HDL: high density lipoprotein; BMI: body mass index; WHR: waist-to-hip ratio; HbA1C: hemoglobin A1C; SBP systolic blood pressure; DBP: diastolic blood pressure; WM: weighted median; IVW: inverse variance weighted.
